# Deciding when to exit

**DOI:** 10.7554/eLife.66591

**Published:** 2021-02-12

**Authors:** Joy H Meserve, Robert J Duronio

**Affiliations:** 1 Department of Cell and Developmental Biology, Perelman School of Medicine, University of PennsylvaniaPhiladelphiaUnited States; 2 Department of Biology, University of North CarolinaChapel HillUnited States; 3 Department of Genetics, University of North CarolinaChapel HillUnited States; 4 Lineberger Comprehensive Cancer Center, University of North CarolinaChapel HillUnited States; 5 Integrative Program for Biological and Genome Sciences, University of North CarolinaChapel HillUnited States

**Keywords:** cell cycle, quiescence, cell proliferation, g1/g0, cdk sensor, *C. elegans*, Zebrafish

## Abstract

A new imaging approach can distinguish between cells destined to stop proliferating and those committed to re-entering the cell cycle in live animals.

**Related research article** Adikes RC, Kohrman AQ, Martinez MAQ, Palmisano NJ, Smith JJ, Medwig-Kinney TN, Min M, Sallee MD, Ahmed OB, Kim N, Liu S, Morabito RD, Weeks N, Zhao Q, Zhang W, Feldman JL, Barkoulas M, Pani AM, Spencer SL, Martin BL, Matus DQ. 2020. Visualizing the metazoan proliferation-quiescence decision in vivo. *eLife* **9**:e63265. doi: 10.7554/eLife.63265

The development and survival of a multicellular organism relies on cells proliferating by passing through a series of events known as the cell cycle. During the first stage of the cycle (G1), each cell makes an important decision: does it progress to the second stage (S-phase) and replicate its genome in preparation for cell division, or does it exit the cycle and become quiescent (Pardee, 1974; Johnson and Skotheim, 2013)?

Cells usually exit the cell cycle in order to differentiate into the distinct cell types of an organism, such as neurons, muscle or fat (Sun and Buttitta, 2017). If the decision to stop proliferating is somehow disrupted, this can affect the normal development or function of tissues and organs and lead to diseases, such as cancer. However, understanding the signals that control this decision can be challenging because a G1 cell preparing to enter S-phase is essentially indistinguishable from a G1 cell that will become quiescent.

One way to overcome this challenge is to visually monitor proteins that are only active in certain phases of the cell cycle (Sakaue-Sawano et al., 2008; Zielke et al., 2014; Grant et al., 2018). For example, a group of proteins called cyclin-dependent kinases (or CDKs for short), which drive cells into S-phase, are active in G1 but are permanently turned off when cells enter a state of quiescence.

In 2013, a group of researchers used this property of CDKs to distinguish cultured mammalian cells in G1 that were preparing to proliferate from those entering quiescence (Spencer et al., 2013). To do this they engineered a fluorescent reporter protein which sits in the nucleus when CDKs are inactive and moves into the cytoplasm when modified by active CDKs (Figure 1A). The nucleus and cytoplasm are easily distinguishable via time-lapse microscopy, making it possible to determine when CDKs are active in individual cells during G1.

**Figure 1.**
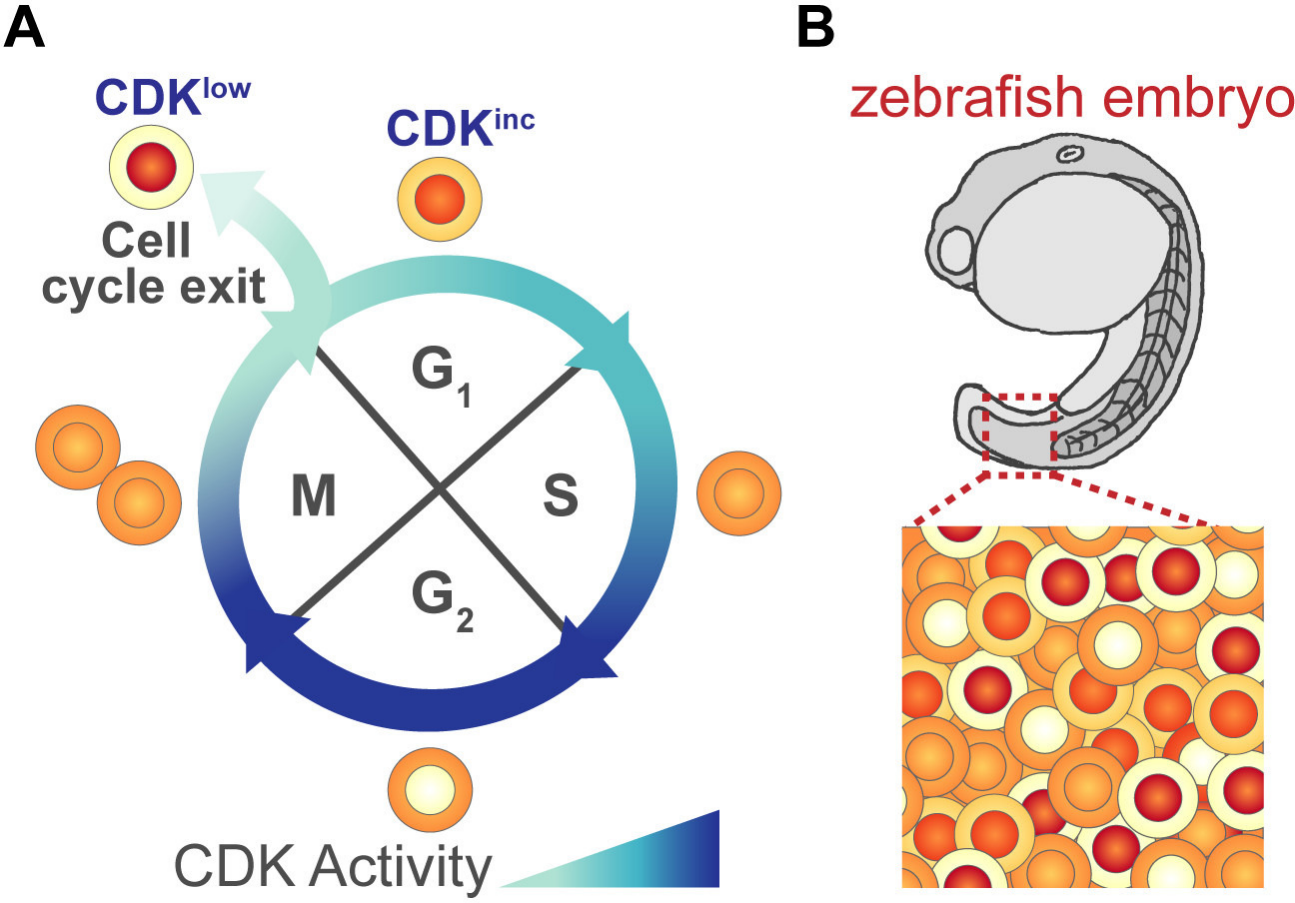
Monitoring cell cycle progression using a fluorescent biosensor. (**A**) As cells grow and prepare to divide they pass through four different phases of the cell cycle: G1, S, G2, M. During the course of this cycle, the activity of cyclin-dependent kinases (CDKs) changes. These fluctuations in activity can be monitored using a fluorescent reporter protein that contains a portion of human DNA helicase B (DHB), which moves from the nucleus to the cytoplasm when phosphorylated by active CDKs. Thus, changes in the levels of DHB in the nucleus and cytoplasm (depicted in shades of orange/red) can be used to determine a cell’s CDK activity. At the start of G1, CDK activity is low and DHB remains in the nucleus (shown in red). Some G1 cells maintain this low level of activity (CDK^low^) and exit the cell cycle to become quiescent, while cells with increasing levels of CDK activity (CDK^inc^) commit to another cell cycle and enter S-phase. (**B**) Adikes et al. showed that the DHB reporter could monitor cell cycle progression of individual cells in live animals, such as the embryo of a zebrafish. It can also identify which cells have exited the cell cycle and which are preparing for division.

Image credit: Joy H Meserve.

Now, in eLife, David Matus from Stony Brook University and co-workers – including Rebecca Adikes, Abraham Kohrman, and Michael Martinez as joint first authors – report how they used this technique to visualize when individual cells decide to stop dividing in living animals (Adikes et al., 2020). To adapt the CDK reporter to animals, the team (who are based at Stony Brook, the University of Colorado, Stanford University, Imperial College and University of Virginia) turned to two well studied experimental organisms: the nematode worm *C. elegans* and the zebrafish *D. rerio*.

Previous work using the CDK reporter in cultured mammalian cells showed that not all G1 cells behave the same after cell division: some never activate CDKs and enter quiescence (CDK^low^ cells), while others begin increasing CDK activity during G1 (CDK^inc^ cells) and ultimately commit to another cell cycle (Figure 1A; Spencer et al., 2013). Adikes et al. found that this bifurcation in G1 cells could also be detected in the tissues of living *C. elegans* and *D. rerio* (Figure 1B). They found that the CDK^low^ and CDK^inc^ phenotypes of G1 cells could be used to predict whether a cell would enter quiescence or would re-commit to another cycle. Further experiments revealed that if a cell had high levels of a protein called p21, which inhibits the activity of CDKs, its daughter cells were more likely to become quiescent following division. This suggests that the decision to proliferate or exit the cell cycle may depend on how p21 levels are regulated in proliferating cells (Overton et al., 2014; Hsu et al., 2019).

The CDK reporter has a number of applications. It could make it easier to study how quiescence is regulated in tissues that are typically difficult to image for long periods of time. It might reveal early steps in tissue regeneration when cells are ramping up to re-enter the cell cycle. It could also be used to sort and recover populations of CDK^low^ and CDK^inc^ cells for further experiments to identify the pathways regulating entry into quiescence.

The CDK reporter will allow us to tackle many interesting questions in developmental biology. For instance, how might the organization of a tissue influence the decision to stop dividing and enter quiescence? Is it possible to identify cells very early in the differentiation process before genes that demark differentiation turn on? Whatever the application or research question, Adikes et al. demonstrate once again that important new insights into the complexities of biology arise when new tools are developed to visualize living organisms.
